# B7-H6, an immunoligand for the natural killer cell activating receptor NKp30, reveals inhibitory effects on cell proliferation and migration, but not apoptosis, in cervical cancer derived-cell lines

**DOI:** 10.1186/s12885-020-07608-4

**Published:** 2020-11-10

**Authors:** Nehla Banu, Annie Riera-Leal, Jesse Haramati, Pablo Cesar Ortiz-Lazareno, Sandeep Surendra Panikar, Blanca Estela Bastidas-Ramirez, Gloria Yareli Gutierrez-Silerio, Fabiola Solorzano-Ibarra, Martha Cecilia Tellez-Bañuelos, Jorge Gutierrez-Franco, Miriam Ruth Bueno-Topete, Ana Laura Pereira-Suarez, Susana del Toro-Arreola

**Affiliations:** 1 Instituto de Enfermedades Crónico Degenerativas, Departamento de Biología Molecular y Genómica, CUCS, Universidad de Guadalajara, Sierra Mojada # 950, Colonia Independencia, CP, 44340 Guadalajara, Jalisco Mexico; 2 Laboratorio de Inmunología, Departamento de Fisiología, CUCS, Universidad de Guadalajara, Guadalajara, Mexico; 3 Institute for Regenerative Cures, Department of Dermatology, University of California-Davis, Davis, USA; 4 Laboratorio de Inmunobiología, Departamento de Biología Celular y Molecular, CUCBA, Universidad de Guadalajara, Guadalajara, Mexico; 5 División de Inmunología, CIBO, Instituto Mexicano del Seguro Social, Guadalajara, Mexico; 6 Centro de Física Aplicada y Tecnología Avanzada, Universidad Nacional Autónoma de México, Querétaro, Mexico; 7 Unidad Académica de Ciencias Químico Biológicas y Farmacéuticas, Universidad Autónoma de Nayarit, Tepic, Mexico

**Keywords:** Cervical cancer, B7-H6, B7H6, Cell proliferation, Cell migration, Apoptosis

## Abstract

**Background:**

Although great progress has been made in treatment regimens, cervical cancer remains as one of the most common cancer in women worldwide. Studies focusing on molecules that regulate carcinogenesis may provide potential therapeutic strategies for cervical cancer. B7-H6, an activating immunoligand expressed by several tumor cells, is known to activate NK cell-mediated cytotoxicity once engaged with its natural receptor NKp30. However, the opposite, that is, the effects in the tumor cell triggered by B7-H6 after interacting with NKp30 has not yet been well explored.

**Methods:**

In this study, we evaluated the surface expression of B7-H6 by flow cytometry. Later, we stimulated B7-H6 positive cervical cancer derived-cell lines (HeLa and SiHa) with recombinant soluble NKp30 (sNKp30) protein and evaluated biological effects using the impedance RTCA system for cell proliferation, the scratch method for cell migration, and flow cytometry for apoptosis. Cellular localization of B7-H6 was determined using confocal microscopy.

**Results:**

Notably, we observed that the addition of sNKp30 to the cervical cancer cell lines decreased tumor cell proliferation and migration rate, but had no effect on apoptosis. We also found that B7-H6 is selectively maintained in tumor cell lines, and that efforts to sort and purify B7-H6 negative or positive cells were futile, as negative cells, when cultured, regained the expression of B7-H6 and B7-H6 positive cells, when sorted and cultivated, lost a percentage of B7-H6 expression.

**Conclusions:**

Our results suggest that B7-H6 has an important, as of yet undescribed, role in the biology of the cervical tumor cells themselves, suggesting that this protein might be a promising target for anti-tumor therapy in the future.

## Background

Cervical cancer is the fourth most common cancer in women worldwide, accounting for an estimated 570,000 new cases and 311,000 deaths in 2018 [1]. The main risk factor for the induction of cervical cancer is high-risk human papillomavirus (HR-HPV) infection [2, 3]. HR-HPV includes 16, 18, 31, 33, 35, 39, 45, 51, 52, 56, 58, 68, and 59, among which type 16 and type 18 are the most prevalent genotypes, causing about 70% of all invasive cervical cancer in the world [4]. Cervical cancer develops from pre-existing non-invasive squamous precursor lesions leading to invasive cervical cancer [5]. These pre-malignant changes range from cervical intraepithelial neoplasia (CIN)1 (mild dysplasia) to CIN2 (moderate dysplasia) to CIN3 (severe dysplasia/carcinoma in situ), representing a spectrum of histological abnormalities [6]. The mortality associated with cervical cancer can be reduced if the disease is detected at the early stages of development or at the pre-malignant stages (CIN 1, 2). For this reason, it is vital to study proteins expressed by transformed cells that may participate in the regulation of the immune response to cervical cancer.

B7 family members are cell-surface protein ligands that are expressed on antigen presenting cells as well as on tumors, which bind to their respective receptors on T lymphocytes and provide positive or negative signals to promote or down-regulate T cell responses [7, 8]. In contrast to the well-known antigen presenting cell expressed B7.1 and B7.2, which provide the crucial second activation signal for T cells [9], B7-H6 is a distinct member of the B7 family that has been shown to be a functional ligand for the NK cell-activating receptor NKp30 that mediates NK cell-dependent killing [10, 11]. This ligand is selectively expressed by tumor cells (such as lymphoma, melanoma, leukemia, and gastric carcinoma), but not by healthy cells, thus making it an important marker and target on tumor cells [12, 13]. Studies have reported altered B7-H6 expression patterns, with upregulation under stress and inflammatory conditions [14]. For example, higher B7-H6 expression has been observed in the skin biopsies of patients with atopic dermatitis [15].

The canonical role described for the B7-H6/NKp30 interaction is activation of the NK cell [16, 17]. At a protein level, it has been found that B7-H6 is selectively expressed by a variety of malignant tumors, such as lymphoma, leukemia [10], gastric carcinoma [18], astrocytoma [19], cervical carcinoma [20] and is also expressed under inflammatory and stress conditions, but has not been seen in healthy cells [14, 21]. Also, high amounts of B7-H6 mRNA have been found in ovarian cancer, brain tumors, breast cancer, and various sarcomas, while normal tissues under steady-state conditions apparently do not show detectable B7-H6 mRNA [22]. Thus, B7-H6 has been reported as a potential prognostic biomarker in breast cancer [23]. In addition, Rusakiewicz et al. showed that higher soluble B7-H6 levels are frequently found in metastatic gastrointestinal stromal tumor patients, compared to patients with localized GIST [24]. The same group showed the clinical impact of the NKp30/B7-H6 axis in high-risk neuroblastoma patients [25]. These features suggest that B7-H6 could be considered as an excellent biomarker for several cancer types.

Based on its structure, the intracytoplasmic domain of B7-H6 has been predicted to contain various signaling motifs, such as an immunoreceptor tyrosine-based inhibitory motif (ITIM), a Src homology 2 domain (SH2), and a Src homology 3 domain (SH3) [12]. These features suggest that upon engagement, B7-H6 might induce a response in the B7-H6^+^ tumor cell. However, the functional relevance of B7-H6 on tumor cells has, as of yet, been poorly explored and deserves future study. Thus, in order to evaluate B7-H6 as a potential target for anti-tumor therapy, it is crucial to obtain an in-depth understanding of its role with regards to tumor proliferation, migration, and apoptosis.

Hence, we hypothesized that B7-H6 might have an unknown inhibitory effect on different biological processes in tumor cells. To prove our hypothesis, we stimulated tumor cell bound B7-H6 ligand using a soluble form of its activating NKp30 receptor and investigated the role of B7-H6 in cervical cancer derived-cell lines (HeLa and SiHa) with respect to proliferation, migration, and apoptosis. We demonstrated that B7-H6 was expressed in HeLa and SiHa cells and when stimulated with recombinant soluble NKp30 (sNKp30) protein, it decreased the proliferation and migration rates and had no effect on apoptosis. Our results suggest that B7-H6 has a wider non-canonical role in tumor biology. Thus, our study has discovered new roles for this molecule on tumor cells and might help to design more effective cervical cancer treatments in the future.

## Methods

### Reagents

Anti-human B7-H6 APC-conjugated antibody, mouse IgG1 APC-conjugated antibody as an isotype control for B7-H6 determination, and recombinant human NKp30 for stimulation of B7-H6 expressing tumor cells were obtained from R&D (Minneapolis, MN, USA). Cisplatin was purchased from Sigma-Aldrich (Merck KGaA). Rabbit anti-human NCR3LG1 polyclonal antibody was purchased from MyBioSource. Mouse monoclonal anti-BAG-6 antibody was obtained from Santa Cruz Biotechnology (Santa Cruz, CA, USA). Alexa Fluor 488-conjugated donkey anti-mouse IgG (H + L) highly cross-adsorbed secondary antibody and Alexa Fluor 594-conjugated goat anti-rabbit IgG (H + L) cross-adsorbed secondary antibody were purchased from Thermo Fisher Scientific, Inc. (Waltham, MA, USA). Fluoro-GEL II mounting medium was obtained from EMS (Hatfield, PA, USA).

### Cell cultures

Human cancer cell lines, HeLa (a uterine cervical adenocarcinoma cell line, positive for HPV 18) and SiHa (a squamous cervical carcinoma cell line, positive for HPV 16) kindly provided by DKFZ, Heidelberg, Germany, were authenticated using the Multiplex Cell Authentication system by Multiplexion GmbH (Friedrichshafen, Germany). The cell lines were tested for mycoplasma contamination using the Universal Mycoplasma Detection Kit (ATCC, Manassas, VA, USA). These cell lines were cultured in complete Dulbecco’s modified Eagle’s medium (DMEM) supplemented with 10% of charcoal-stripped fetal bovine serum, penicillin-streptomycin (10,000 U/mL). DMEM, charcoal-stripped serum, TrypLE™ Express enzyme, and penicillin-streptomycin were obtained from Thermo Fisher Scientific, Inc. (Waltham, MA, USA). Cells were kept in a water-jacketed CO_2_ incubator at 37 °C in an atmosphere containing 5% CO_2_; cells were grown without exceeding 80% confluence.

### Evaluation of cell surface expression of B7-H6

The presence of B7-H6 in HeLa and SiHa cells were determined by flow cytometry. Parallel sets of experiments were performed using single-cell suspensions of 500,000 cells per tube. From which, one set was stained with anti-human B7-H6 APC-conjugated antibody, another with an isotype control (mouse APC-conjugated IgG1). Cells were examined using a FACS flow cytometer (FACSCalibur; BD Biosciences, San Jose, CA, USA) and files were then further analyzed using FlowJo software (FlowJo LLC; Ashland, OR, USA).

### Cell sorting of B7-H6 positive and B7-H6 negative populations

B7-H6 positive and negative populations were sorted using FACS. The sorted populations were cultured for two weeks under the above-mentioned conditions. Later, the surface expression of B7-H6 in each population was determined by flow cytometry.

### Cell proliferation

The proliferation rate of HeLa and SiHa cells bearing B7-H6 was assessed using the xCELLigence platform (Roche Applied Science, Penzberg, Germany), which is based on real-time monitoring of cell properties, such as cell adherence and proliferation. Five thousand cells were cultured using DMEM medium in the xCELLigence reading station and were allowed to adhere. After 4 h, cells were stimulated with different concentrations of recombinant human sNKp30 (0, 5, 10, 20, 40, 60, and 80 ng/mL were added to the respective wells). The proliferation rate was assessed in real-time every 30 min over a period of 96 h. Cisplatin stimulation was used as a control for the induction of cell death. The proliferation rate was derived from the cell index of the RCTA plate and xCELLigence platform. The cell index was calculated as the difference between the impedance of a well with only cells minus the impedance of a well with only culture media, divided by the nominal impedance of the system. There is a direct correlation between the number of cells attaching and the cell index readout on the machine. Our statistical analysis was based on comparisons between the cell index values of the controls and each experimental condition. Three independent experiments were performed with six replicates in each case.

### Wound-healing assay

In order to analyze the influence of B7-H6 on cell migration, we utilized a wound-healing assay. In brief, tumor cells were seeded in 6-well plates at a density of 0.1 × 10^6^ cells/3 mL/well. Once the cells reached approximately 80% confluence, a single scratch was performed using a sterile 10 μL pipette tip. Then, the medium with the floating dead cells was replaced with fresh medium in each well. Later, cells were stimulated with different concentrations of recombinant human sNKp30 (0, 20, 40, 60, and 80 ng/mL) in the respective wells. The extent of wound closure was measured every 24 h after wounding.

### Cell apoptosis analysis

Cell apoptosis was determined by flow cytometry using the APC Annexin V apoptosis detection kit with 7-AAD from Biolegend. Briefly, tumor cells were plated onto 6-well plates at a concentration of 0.1 × 10^6^ cells/3 mL/well. After 24 h of incubation, cells were stimulated with different concentrations of recombinant human sNKp30 (0, 20, 40, and 80 ng/mL). Then, after 48 h, the percentage of early apoptotic, late apoptotic, and dead/necrotic cells was determined using the Annexin V kit according to the manufacturer’s instructions. The data from three independent experiments were analyzed using FlowJo software and reported as geometric mean fluorescence intensity (MFI).

### Localization of NKp30 ligands

The localization of NKp30 ligands B7-H6 and BAG-6 was performed by staining with their respective antibodies (as described above) and confocal microscopy observation. In these experiments, the cells were fixed with 4% formaldehyde for 10 min followed by permeabilization with Tween 20 at 0.2% for 10 min at room temperature. Fixed cells were blocked with 1% BSA along with 10% bovine fetal serum in PBS and then incubated overnight at 4 °C with primary antibodies (rabbit anti-human B7-H6 polyclonal antibody and mouse anti-human BAG-6 monoclonal antibody with the dilution of 1:1000 and 1:100 in PBS, respectively) After being washed with PBS, cells were stained with the corresponding Alexa Fluor 488- and Alexa Fluor 594-conjugated secondary antibodies for 60 min at room temperature. Finally, cells were washed with PBS and were mounted with Fluoro-Gel mounting medium with a counter stain for DNA (DAPI). Confocal microscopy was used to take the images of these stained cells. The confocal images were analyzed using a Leica TCS SPE confocal microscope equipped with a Leica DFC 365 FX Digital Camera, and a filter-free tunable spectral detector (430–750 nm). The objective lenses used was 63X oil immersion. For Alexa Fluor 488-conjugated secondary antibodies, the images were reached at an excitation of 490 nm and the emission signal collected at 525 nm; for Alexa Fluor 594-conjugated secondary antibodies, the excitation was 590 nm and emission at 617 nm. Images were captured and managed through the X® LAS (Leica Microsystems) format software. All the images were acquired with a resolution of 650 × 650 pixels and used without any downstream processing or averaging; threshold manipulations, expansion or contraction of signal ranges and the altering of high signals were strictly avoided. No channel or signal adjustments in the merged images were made.

### Statistical analysis

Statistical assessment was conducted using SPSS Statistics 20 (IBM Corp., Armonk, NY, USA) and GraphPad Prism 6 software (GraphPad Software, Inc., La Jolla, CA, USA). Data obtained from three independent tests were analyzed using a one-way ANOVA in SPSS and are presented as means ± standard error; *p*-value ≤0.05 was considered to indicate a statistically significant difference.

## Results

### Cell surface expression of B7-H6 in cervical cancer-derived cell lines

**Fig. 1:**
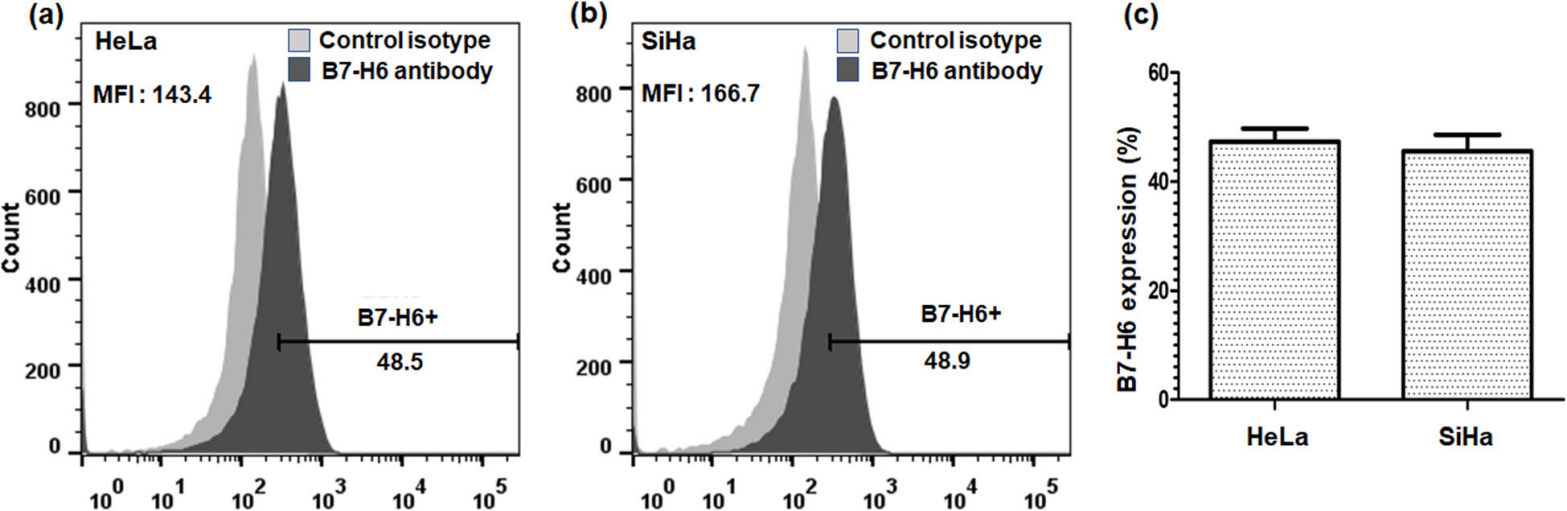
Surface expression of B7-H6 in HeLa and SiHa cells after 48 h culture. B7-H6 expression was analyzed by flow cytometry. HeLa and SiHa cells were stained with human B7-H6 APC-conjugated antibody (black histograms) or mouse IgG1 APC-conjugated isotype control (gray histograms). **a** MFI of HeLa cells. **b** MFI of SiHa cells. **c** Percentage of B7-H6 positive HeLa and SiHa cells in each cell culture. Data are representative of at least 3 independent experiments

To investigate the effect of B7-H6 stimulation on cervical cancer-derived cell lines HeLa and SiHa, we first measured the cell surface expression of B7-H6 in these two cell lines by flow cytometry. For this purpose, three independent experiments were performed. In all experiments, HeLa cells, which are derived from adenocarcinoma of the cervix, expressed B7-H6 at around 48.5%, meanwhile SiHa cells (squamous cell carcinoma), similarly expressed B7-H6 at around 48.9%, on the cell surface as shown in Fig. 1a and b, respectively. This experiment was repeated three times with similar results, with no significant difference found between the expression of B7-H6 in HeLa or Siha cells, Fig. 1c.

### Cell surface expression of B7-H6 in cervical cancer-derived cell lines after cell sorting of B7-H6 positive and negative populations

**Fig. 2:**
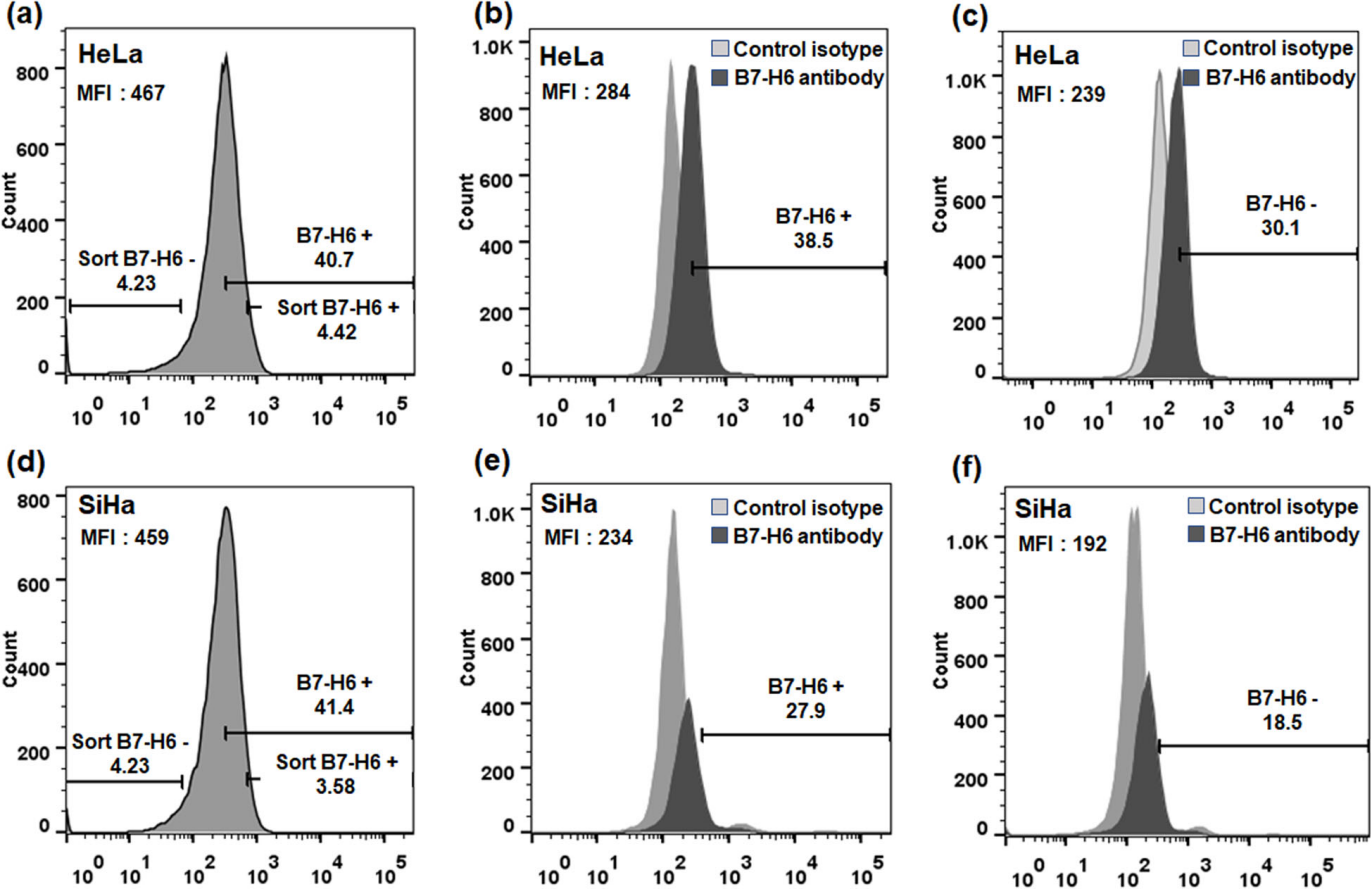
Surface expression of B7-H6 in sorted populations cultured for two weeks. B7-H6 positive and B7-H6 negative HeLa or SiHa populations were sorted and cultured for two weeks. B7-H6 expression was analyzed by flow cytometry. In (**a**) and (**d**): HeLa or SiHa, respectively, cells at the beginning of sorting; cells were both positively and negatively sorted as is reflected by the region markers showing the gates used to define these two populations. In (**b**) and (**e**): the resulting B7-H6 MFI and percentage of B7-H6 positively sorted cells after two weeks of culture. In (**c**) and (**f**): the resulting B7-H6 MFI and percentage of B7-H6 negatively sorted cells after two weeks of culture. Black histograms represent the B7-H6 antibody conjugated with APC, while the grey histogram represents mouse IgG1 APC-conjugated isotype control. Data are representative of at least 3 independent experiments

After determining the surface expression of B7-H6 in HeLa and SiHa cells, we attempted to identify pure sorted B7-H6 positive and B7-H6 negative populations based on FACS as shown in Fig. 2a and d. After culturing the sorted cells for two weeks, we again evaluated the expression of B7-H6 in the respective populations. Results from HeLa cells surprisingly revealed that the expression of B7-H6 in the sorted B7-H6 positive population was only observed in 38.5% of the cells and not in near totality as expected; on the other hand, B7-H6 expression in the sorted B7-H6 negative population was present in 30.1% of these cells, as shown in Fig. 2b and c. With respect to SiHa, similar behavior was also seen; that is, B7-H6 expression in the sorted B7-H6 positive population was observed to be 27.9%; while the expression in the sorted B7-H6 negative population was 18.5%, as shown in Fig. 2e and f. These data indicate that a certain percentage of the positive population had lost the B7-H6 expression, while the negative population had regained the expression of B7-H6 in both cell lines; thus, cell surface expression of B7-H6 appears to be transient and selectively maintained at only a percentage of the total cell populations.

### Stimulation of B7-H6 with its natural receptor NKp30 diminishes the proliferation rate of cervical cancer cells

**Fig. 3:**
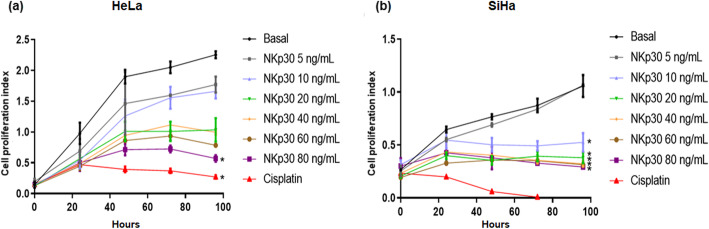
Effect of stimulation of B7-H6 on cell proliferation. Unsorted tumor cell lines were incubated with soluble recombinant NKp30 receptor. Proliferation was determined using the xCELLigence RTCA platform. In (**a**) HeLa, and (**b**) SiHa, the cells were stimulated with different concentrations of sNKp30 (5, 10, 20, 40, 60 and 80 ng/mL) and were compared with the basal (cells incubated only with media, 0 ng/mL NKp30). Cisplatin (1 μg/mL) was used as a control for the inhibition of proliferation. Proliferation was evaluated for 96 h. The graphs reflect the results of three independent experiments performed in sextuplicate; *p < 0.05 (analysis of variance)

Subsequently, the effect of B7-H6 stimulation through its natural ligand NKp30 on cervical cancer cell proliferation was investigated by the xCELLigence real-time cell analysis (RTCA) system, which can continuously monitor cell proliferation. For this purpose, the cervical cancer-derived cell lines HeLa and SiHa were stimulated with recombinant human sNKp30 at different concentrations (0, 5, 10, 20, 40, 60, and 80 ng/mL). The proliferation rate was measured every 24 h until 96 h. Cisplatin (1 μg/mL) was used as a control for the inhibition of tumor cell proliferation. As shown in Fig. 3a, HeLa cells showed a decrease in their proliferation index when compared to basal, when stimulated with the above-mentioned concentrations; however, a significant difference was only seen when sNKp30 was added at a concentration of 80 ng/mL. SiHa cells in turn, as shown in Fig. 3b, showed a significant decrease in their proliferation index when compared to basal, when stimulated with 10, 20, 40, 60, and 80 ng/mL of sNKp30. Altogether, these data indicate that stimulation of B7-H6 significantly diminished the proliferation of cervical cancer derived-cell lines, in a time-dependent manner, once incubated with its receptor, NKp30.

### Stimulation of B7-H6 diminishes the migration potential of cervical cancer-derived cell lines

**Fig. 4:**
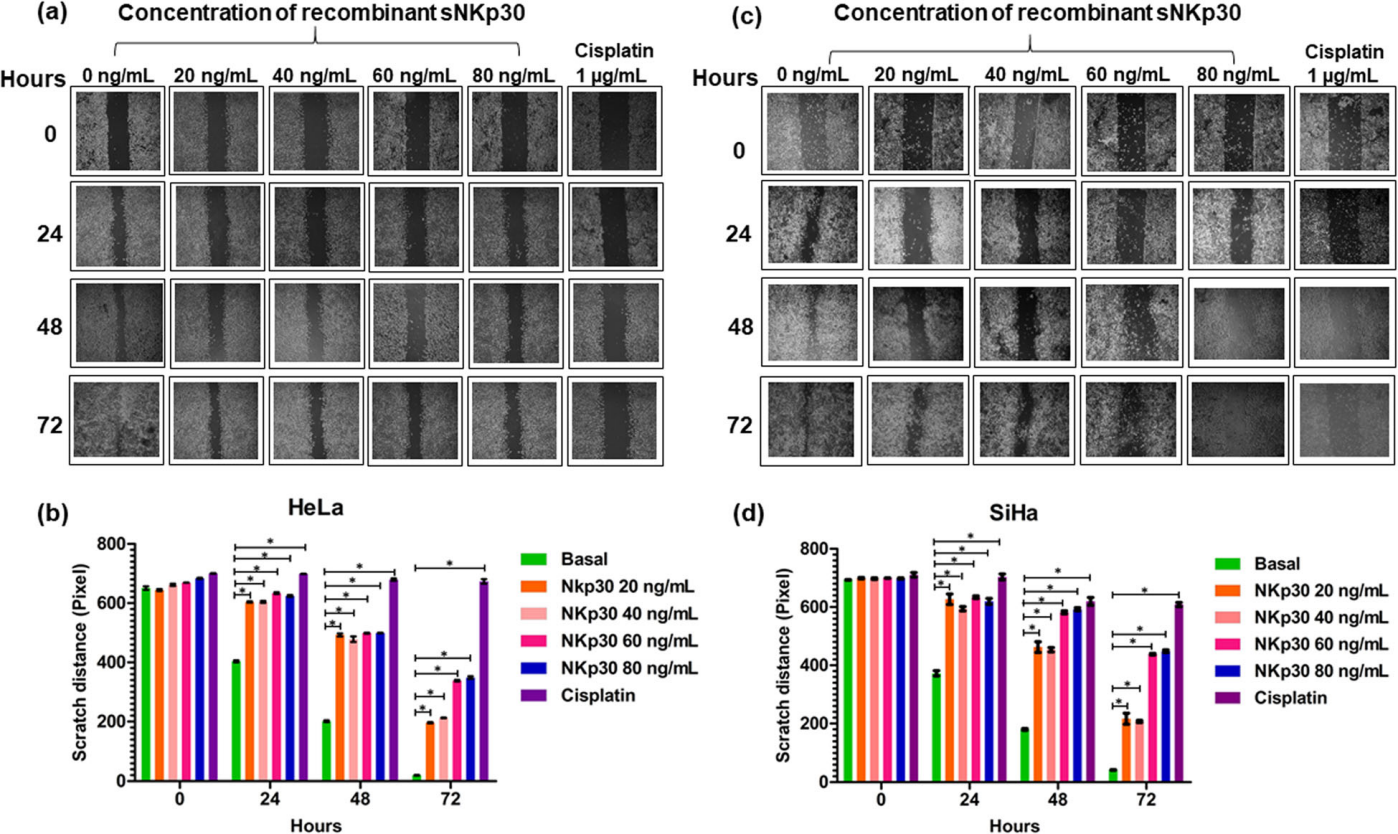
Effect of stimulation of B7-H6 on tumor cell migration. Using the scratch method to approximate cell migration or wound healing, the effect of B7-H6 stimulation was evaluated for 72 h. In (**a**) and (**c**): photographs of unsorted HeLa and SiHa cells migrating across cell free “wounds” in culture. Cells were incubated with 0–80 ng/mL of sNKp30 and compared with cells incubated with media (basal) or cisplatin (1 μg/ml), a control for the inhibition of migration. In (**b**) and (**d**): graphical representation of the resulting changes in scratch distance (wounds) from three independent experiments with HeLa and SiHa cells, respectively; *p < 0.05 (analysis of variance)

To further evaluate the effect of B7-H6 stimulation on the migration potential of cervical cancer-derived cell lines HeLa and SiHa, wound healing assays were performed. Tumor cells were stimulated with different concentrations of recombinant sNKp30 (0, 20, 40, 60, and 80 ng/mL). Cisplatin at 1 μg/mL was used as a positive control for the inhibition of cell migration. The extent of wound closure was measured every 24 h up to 72 h after wounding as shown in Fig. 4a and c. Both HeLa and SiHa cells showed significant differences between basal and all of the above-mentioned concentration of sNKp30, as shown in Fig. 4b and d. Similar to the proliferation rate assay, these data indicate that stimulation of B7-H6 significantly diminished the migration potential of cervical cancer-derived cell lines, once incubated with its receptor, NKp30.

### Stimulation of B7-H6 did not show an effect on apoptosis induction in cervical cancer-derived cell lines

**Fig. 5:**
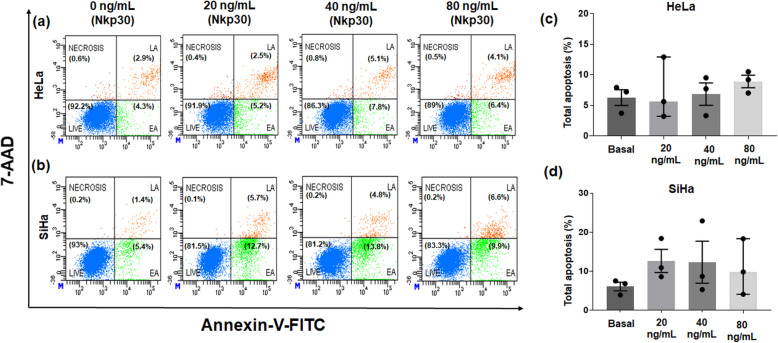
Effect of stimulation of B7-H6 on tumor cell apoptosis. Apoptosis was evaluated using Annexin V-FITC/7-AAD staining and analyzed by flow cytometry. (**a**) HeLa cells and (**b**) SiHa cells were stimulated with different concentrations of sNKp30 (20, 40, 80 ng/mL) and were compared with the basal treatment (NKp30 0 ng/mL). In (**c**) and (**d**): graphical results of three independent experiments with HeLa and SiHa cells, respectively; *p < 0.05 (analysis of variance)

As mentioned in the Methods section, Annexin V-FITC/7-AAD staining was examined by flow cytometry to investigate the induction of apoptosis in HeLa and SiHa cells after stimulation of B7-H6 by addition of sNKp30. Cell apoptosis was defined as Annexin V^+^ (early apoptosis) or Annexin V^+^/7AAD^+^ (late apoptosis). Although the addition of sNKp30 at different concentrations (20, 40, and 80 ng/mL) showed a smooth increase in apoptosis induction when compared with basal (no addition of sNKp30) as seen in Fig. 5a and b, we did not observe any significant result in either of the cell lines (Fig. 5c and d). This result suggests that B7-H6 may have no role in the apoptotic pathway, at least under these conditions.

### Localization of NKp30 ligands in cervical cancer-derived cell lines

**Fig. 6:**
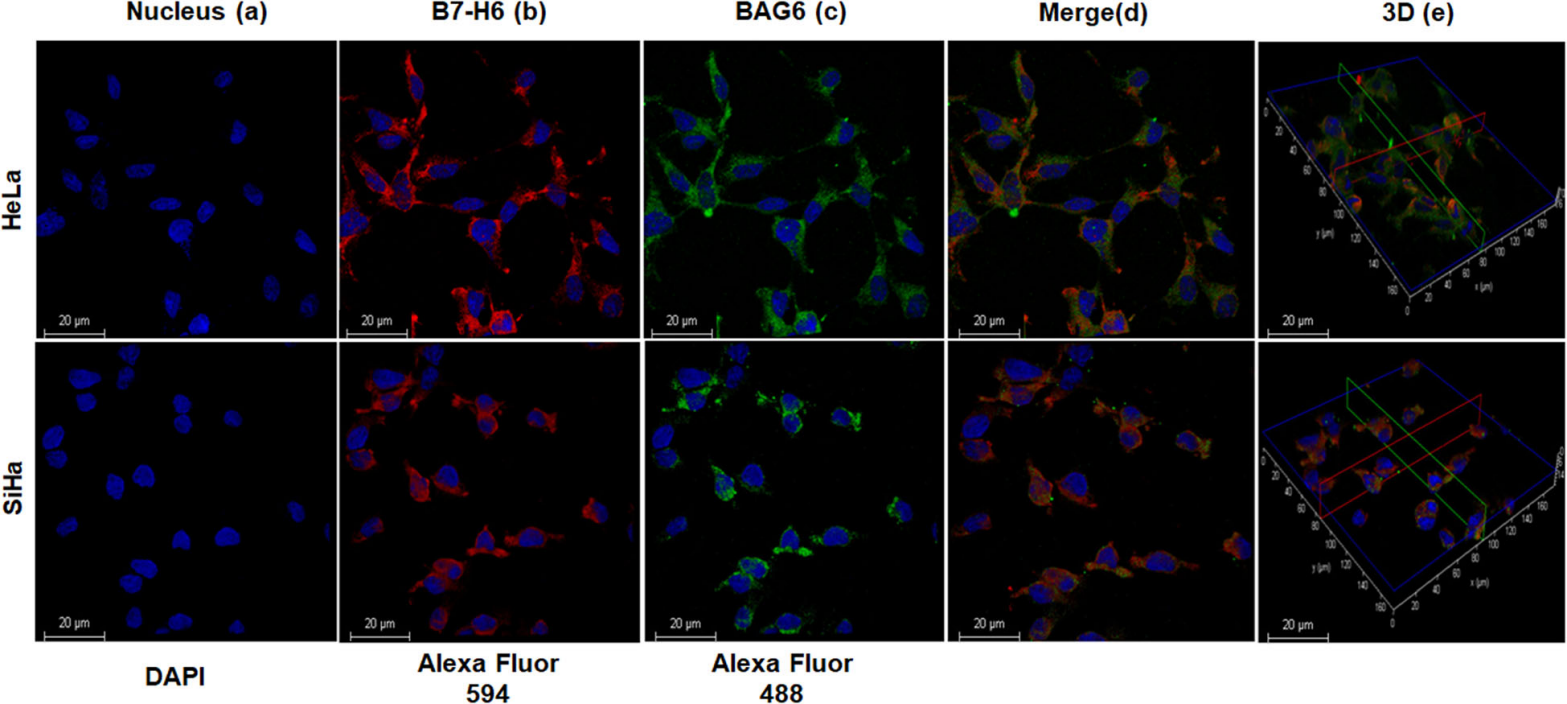
Localization of NKp30 ligands in tumor cells. Confocal microscopy was performed to show B7-H6 in red (**b**) and BAG-6 in green (**c**); the nucleus was counterstained with DAPI in blue (**a**). The upper panel shows HeLa while the lower panel shows SiHa. The merge column (**d**) shows that expression of B7-H6, while similar, is distinct from BAG-6; and (**e**) represents the 3-dimentional images of the same

While currently B7-H6 has been well established as an activating ligand for NKp30, initially a different molecule, BAG-6, a BCL-2 associated anti-apoptotic protein associated with tumor cells, was considered to be the only activating ligand for NKp30 [26, 27]; in 2009 Brandt et al. demonstrated that B7-H6, another surface molecule on tumor cells, could bind and activate NKp30 [10]. Because in our experiments we were using soluble NKp30, we wanted to disregard the possibility that we were binding BAG-6. Both NKp30 ligands were localized predominantly to the cytoplasm and to a lesser extent to the cell surface, as observed in Fig. 6a (DAPI as a nuclear counterstain), 6b and Fig. 6c. Interestingly, both ligands were localized at very near proximity to each other on the individual cells, although they were not colocalized, as can be observed after merging the images as shown in Fig. 6d. When compared to BAG-6, B7-H6 was observed with higher expression in both cell lines. Three-dimension images of the staining (Fig. 6e) further confirmed our result, that the staining pattern was distinct.

## Discussion

We showed that the surface expression of B7-H6 was present in around 50% of the cells in both cervical cancer cell lines, HeLa and SiHa. We are among the first to show the presence of B7-H6 in these cell lines, and these results are in line with a recent report by our group, where the expression of B7-H6 was consistently seen in cervical cancer specimens [20] As the expression of B7-H6 was not present in the totality of HeLa or SiHa cells, we tried to sort the B7-H6 positive and B7-H6 negative population and found that B7-H6 was selectively maintained or lost in the tumor cell lines. Both the positive and negative cell lines regained the expression with roughly the same percentage. We are the first to note this type of selective expression of this molecule in Hela and SiHa cells. Hence, it is vital to explore the regulation of B7-H6 in tumor cells. Though some studies show down-regulation of B7-H6 by metalloproteases, such as ADAM-10 or ADAM-17, [28] and histone deacetylases (HDACs) [29], which could explain in part the loss of B7-H6 in some of the cells, the gain of B7-H6 expression in the negative population is not clear yet. Nor is the apparent selective pressure to maintain only about 20–30% B7-H6 expression after two weeks of culture in both positive and negatively selected B7-H6 groups. We have not ruled out that B7-H6 could be regulated by cell cycle proteins; the cell cycle has been found to control the expression of many activating NK ligands for NCRs (natural cytotoxicity receptors) and NKG2D [30] and indeed an early paper working with the as of then unidentified NKp30L, reported that this ligand was down-modulated in HEK 293 cells arrested in G2/M phase after colchicine treatment [31].

Based on the above, the remaining sets of experiments in the project were performed using the total population of HeLa and SiHa cells, and not in sorted populations as had initially been planned.

Proliferation and migration exemplify some of the main hallmarks of cancer [32]. Structural studies of B7-H6 have predicted various signaling motifs in the intracytoplasmic domain of B7-H6, such as an immunoreceptor tyrosine-based inhibitory motif (ITIM), an Src homology 2 (SH2)-binding domain, and an Src homology 3 domain (SH3) [33]. These observations suggested that upon interaction with NK cells, B7-H6 not only mediates NK cell-dependent killing, but may also trigger some signaling pathways within the tumor cell itself, potentially inducing changes in the behavior of the tumor cells. To perhaps explain these changes, it is useful to return to the cytoplasmic domain of B7-H6. When ITIM-containing receptors become tyrosine phosphorylated, there is a recruitment of SH2 domain-containing phosphatases, which inhibit cell activation [34]. Here, we have shown that proliferation and migration of HeLa and SiHa cells were significantly decreased after stimulating with different concentrations of sNKp30. This result might suggest that the presence of ITIM-bearing B7-H6 on the cervical tumor cell leads to an interaction with the NK cells that both activates the NK cells and, after interacting with its natural receptor NKp30, transduces an intracellular signal leading to a decrease in the proliferation and migration rate of the B7-H6 presenting tumor cell. It is important to also note that despite similar surface percentages of B7-H6, HeLa and SiHa cells behaved very differently with respect to proliferation. We suspect the different cell culture dynamics are mostly at play here. Hela normally grows much faster and thus may be less affected by sNKp30 binding. Even when we add cisplatin to Hela and SiHa cells, we observed that SiHa is more sensitive to cisplatin than HeLa.

In contrast to our findings on proliferation and migration, other studies have found different results. For instance, Fenguan et al. reported that proliferation, migration, and invasion of glioma cells were inhibited after knocking down B7-H6 [35]. Bing et al. demonstrated that the knock down of B7-H6 inhibited cell proliferation and migration, and promoted apoptosis in triple-negative breast cancer [36]. Additionally, Feifei et al. demonstrated that the knock down of B7-H6 also inhibited cell proliferation, colony formation and migration/invasion of lymphoma cells [37]. However, all the above-mentioned studies on the effects of B7-H6 with respect to proliferation, apoptosis or migration are knock down studies. It might be possible that the tumor may express other NKp30 ligands, such as galactin 3, BAG-6, or others [26]; thus, there exists the possibility that the results in those B7-H6-knocked down cancer cells might be due to compensatory over-expression of other ligands, or other tumor: NK cell interactions. For this reason, we performed confocal microscopy in an attempt to reveal the localization of the NKp30 ligands B7-H6 and BAG-6, in both HeLa and SiHa cells. Our results here were not conclusive; while we found higher expression of B7-H6 as compared to BAG-6, we still saw significant BAG-6 expression on the cell membrane. To help explain this we turn to the literature: in one of the B7-H6 knock down studies, the authors concluded that, based on the overwhelming observed abrogation of NKp30 activation-induced responses when siRNA was used, that the other proposed NKp30 ligands had to have been nonrelevant, nonfunctional, or so minimally functional as to be below the level of detection in their system [38]. In line with this idea, BAG-6 has been reported to be either nuclear, or released in exosomal vesicles [39], thus the apparent cell surface staining that we observed might have been only transient.

Returning to what was touched on above, the potential mechanism behind our observations: ITIM sequences are common in the cytoplasmic tails of inhibitory receptors. A famous example is the case of the inhibitory KIRs, the killer-cell immunoglobulin-like receptor family found in NK cells. These inhibitory KIRs have ITIM sequences in their cytoplasmic domains. Upon tyrosine phosphorylation, these ITIMs are able to recruit SH2-containing tyrosine phosphatases (SHP)-2 in order to mediate their inhibitory function [40]. The B7-H6 cytoplasmic domain also contains predicted ITIM sequences, as mentioned above, suggesting that B7-H6 might also function as an inhibitory molecule. However, this raises the question, why would a tumor cell experience selective pressure to upregulate this ligand? One possible answer arises when analyzing the tumor microenvironment and other factors that may be at play. The tumor environment is characterized by chronic local inflammation and, among other factors, over production of metalloproteases. Interestingly, knock down of B7-H6 has been linked to decreased production of MMP-2 and MMP-9 [35]. Perhaps even more interestingly, expression of metalloproteases has been linked to shedding of B7-H6 [28]. This shedding, similar to what has been observed with other NK cell activating ligands such as MICA/B:NKG2D [41], might lead to receptor endocytosis and down-regulation in cytotoxic NK cells [42], thus inhibiting the antitumor response. In the future we will have to characterize the environment around tumor cells in situ, and correlate the expression of B7-H6 with clinicopathological features and overall survival in cervical cancer. Perhaps cervical cancer cells express B7-H6, even at obvious costs to their survival (inhibition of proliferation and migration) with the trade-off that high expression of B7-H6 leads to increased soluble B7-H6, which is a viable anti-NK cell immune evasion mechanism.

Finally, it will be interesting to perform future work with other cell lines that express higher levels of B7-H6. Principally, cells lines (or cells from tumor samples in patients) that express very high levels of B7-H6 would be expected to more readily activate NK cells, and, obversely, would be expected to show more clearly the results that we report here, namely, inhibition of proliferation and migration. It will be an interesting question as to whether a greater density of tumor-side B7-H6 signaling would lead to significant changes in apoptosis, which we did not observe here. Additionally, it will be important to measure the relationship between B7-H6 expression and soluble B7-H6 liberation, and their effects (both positive and negative, potentially) on NK cell activation.

## Conclusions

Our current results show that B7-H6, a biomarker in several malignant tumors, was expressed by the cervical cancer-derived cell lines, HeLa and SiHa. Stimulation of HeLa and SiHa cells with soluble NKp30 showed a decrease in proliferation and migration processes and had no functional effect on apoptosis. This finding suggests that the interaction of B7-H6 with NKp30 not only mediates NK cell-dependent cytotoxic killing of tumor cells, but might also mediate signaling cascades, possibly through ITIM, SH2, and SH3 in B7-H6, resulting in decreased tumor cell proliferation and migration. However, further studies are required to elucidate the underlying mechanisms of how this ligand inhibits tumor cell proliferation and migration by studying the intracellular motifs of B7-H6.

## Data Availability

The datasets used and/or analyzed during the current study are available from the corresponding author on reasonable request.

## Abbreviations

NKp30: Natural Killer p30
ITIM: Immunoreceptor tyrosine-based inhibitory motif
SH2: Src homology 2 domain
SH3: Src homology 3 domain
HR-HPV: High-risk human papillomavirus
ITAM: Immunoreceptor tyrosine-based activation motif
